# DAX-1 Expression in Pediatric Rhabdomyosarcomas: Another Immunohistochemical Marker Useful in the Diagnosis of Translocation Positive Alveolar Rhabdomyosarcoma

**DOI:** 10.1371/journal.pone.0133019

**Published:** 2015-07-13

**Authors:** Calogero Virgone, Enzo Lalli, Gianni Bisogno, Elena Lazzari, Josep Roma, Angelica Zin, Elena Poli, Giovanni Cecchetto, Patrizia Dall’Igna, Rita Alaggio

**Affiliations:** 1 Pediatric Surgery, Department of Women’s and Children’s Health, University-Hospital of Padua, Padua, Italy; 2 Institut de Pharmacologie Moléculaire et Cellulaire, Unité Mixte de Recherche 7275, CNRS, Valbonne, France; 3 Université de Nice–Sophia Antipolis, Valbonne, France; 4 Hematology Oncology, Department of Women’s and Children’s Health, University-Hospital of Padua, Padua, Italy; 5 Pathology Unit, San Bortolo Hospital, Vicenza, Italy; 6 Laboratory of Translational Research in Pediatric Cancer, Vall d’Hebron Research Institute, Universitat Autònoma de Barcelona, Barcelona, Spain; 7 Istituto della Ricerca Pediatrica "Città della Speranza", Laboratorio di Biologia dei Tumori Solidi, Padova, Italy; 8 Pathology Unit, Department of Medical and Diagnostic Sciences and Special Therapies, University of Padua, Padua, Italy; University Hospital of Modena and Reggio Emilia, ITALY

## Abstract

**Objectives:**

The aim of this study was to investigate the expression of DAX-1 in a series of pediatric rhabdomyosarcomas (RMS) with known translocation and compare it to Ap2β, known to be selectively expressed in ARMS.

**Design:**

We revised a series of 71 alveolar rhabdomyosarcomas (ARMS), enrolled in the Italian Protocols RMS 79 and 96, and 23 embryonal rhabdomyosarcomas (ERMS) as controls. Before investigating Ap2β and DAX-1, ARMS were reviewed and reclassified as 48 ARMS and 23 non-ARMS.

**Results:**

Translocation positive ARMS showed a characteristic Ap2β/DAX-1+ staining pattern in 78% of cases, while 76% of classic ERMS were negative for both. Ap2β alone was positive in 3.9% of RMS lacking translocation, whereas DAX-1 alone was positive in 25.4%. Conversely, 9% and 6% of translocation positive ARMS were positive only for DAX-1 or Ap2β, respectively. The 23 non-ARMS shared the same phenotype as ERMS but had a higher frequency of DAX-1 expression.

**Conclusions:**

DAX-1 is less specific than Ap2β, however it is a sensitive marker for translocation positive ARMS and can be helpful in their diagnosis if used in combination with Ap2β.

## Introduction

Rhabdomyosarcoma (RMS) is the most common soft tissue sarcoma in children, accounting for about 8% of all pediatric malignant tumors [1]. The current WHO classification recognizes four main histological subtypes with distinctive clinico-pathological features: embryonal rhabdomyosarcoma (ERMS), alveolar rhabdomyosarcoma (ARMS), spindle cells/sclerosing rhabdomyosarcoma and pleomorphic rhabdomyosarcoma [2]. ERMS are more frequent in younger patients, occur mostly in the genito-urinary region or orbit and lack specific recurrent genetic alterations [3,4]. By contrast, ARMS typically occur in older children, are more frequent in the extremities and behave more aggressively. About 80% of ARMS are characterized by recurrent translocations, t(2;13)(q35;q14) or t(1;13)(p36;q14), resulting in 2 different fusion transcripts, *PAX3-FOXO1* and *PAX7-FOXO1* respectively, encoding for proteins that act as aberrant transcription factors [5,6]. Nevertheless, the histological definition of ARMS has been recently under debate [3,7], suggesting that translocation negative ARMS may actually represent a variant of ERMS, characterized by a less aggressive behaviour and better outcome that may support the possibility of a less intensive treatment [7,8,9]. Genomic analysis of RMS plays an important role in redefining their classification and identifying genes selectively expressed in different RMS subtypes. The proteins encoded by some of these genes may be recognized by commercially available antibodies and represent a useful diagnostic tool in clinical practice. In particular, the *TFAP2* gene (Ap2β) is highly expressed in translocation positive ARMS [10,11].

DAX-1 is an orphan nuclear receptor involved in gonadal development, sex determination and steroidogenesis encoded by the *NR0B1* gene. Beside playing an important role in the regulation of stemness, under the control of the Nanog transcription factor in mouse embryonic stem cells [12,13,14], it is also expressed in Ewing sarcoma/primitive neuroectodermal tumor (PNET), in which it is up-regulated by the fusion transcript EWS-FLI1, promoting cell proliferation and inhibiting apoptosis [12,13,15]. DAX-1 positive expression has been reported only in 2 cases of translocation-positive ARMS [13] but it has never been systematically investigated in RMS.

The aim of this study was to investigate the expression of DAX-1 and its potential role as a diagnostic tool comparing it to the expression of Ap2β (encoded by *TFAP2*) in a series of RMS with known translocation.

## Materials and Methods

Ninety-four RMS enrolled in the Italian protocols RMS 79 and RMS 96 were retrieved from the archives of the institutional and consultation files at the Pathology Department of the University of Padua. These included 71 cases diagnosed and treated as ARMS and 23 classic ERMS, which were used as “controls”. HE stained sections, as well as desmin and myogenin immunostains, were reviewed.

The 71 ARMS were reclassified, according to the current WHO classification, as: ARMS, ERMS and spindle cell/sclerosing RMS. The group of RMS with mixed features of ERMS and ARMS, previously classified as ARMS but probably representing a subgroup of ERMS, were classified as mixed RMS, and a new, provisional category of “epithelioid RMS”, recently described by Jo *et al*., [16], was also introduced

Presence of anaplasia was also evaluated, according to the criteria used for Wilms’ tumor [17,18].

In all cases, the diagnosis was then correlated with the results of reverse transcription polymerase chain reaction (RT-PCR) analysis, for *PAX3/PAX7-FOXO1* fusion genes. In 63 cases molecular investigations were performed at diagnosis on frozen tissue, in 8 on paraffin-embedded tissue. RNA extraction was carried out from 4-μm slices. Samples from paraffin-embedded tissue were dewaxed in two changes from d-limonene and washed three times in ethanol (100%, 90% and 70%): rehydrated tissue was then incubated in lysis buffer mixture containing proteinase-K (Absolutely RNA FFPE, Stratagene, Santa Clara, California). After 3–18 h of digestion at 55°C in lysis buffer, the RNA was extracted and then redissolved in 30 μL of RNase-free water elution (10 mM Tris-HCl, pH7.5). Samples from frozen tissue were directly transferred in Eppendorf 1.5 Rnase free: tissue was then incubated in lysis buffer mixture containing proteinase-K. After 3–18 hours of digestion at 55°C in lysis buffer, the RNA was extracted and then redissolved in 30 μL of RNase-free water eluition (10 mM Tris-HCl, pH7.5). 1 μg of RNA was retrotranscribed to cDNA with SuperScript III Reverse Transcriptase (Invitrogen, MILANO). Quantitative RT-PCR for MyoD1, PAX3-FOX01 e PAX7-FOX01 was performed on ABI Prism 7000 (Applied Biosystems, Foster City, California), with Taq-Man technology. The ABL gene was used as positive control. Amplification and detection were performed as follows: 40 cycles with 2 minutes at 50°C; 10 minutes at 95°C, 15 seconds at 95°C, 1 minute at 60°C (S1 Table).

In the cases in which the morphological features were those of a classic ARMS, the diagnosis of ARMS was confirmed, even in the absence of the transcripts.

Immunohistochemical staining was performed on 3 μm formalin-fixed, paraffin-embedded tissue sections using a fully automated system (Bond—maX, Leica, Newcastle Upon Tyne, UK). Sections were dewaxed and rehydrated and incubated in retrieval buffer solution (Leica) for antigen recovery. Specimens were then washed with phosphate-buffered saline (pH 7.0) and incubated with the Bond Polymer Refine Detection Kit (Leica) according to the manufacturer’s protocols. Staining was visualized with 3,3’-diaminobenzidine, and the slides were counterstained with Mayer’s hematoxylin. When not available or poorly preserved, immunostains for desmin (DAKOCytomation, Clone D33) and myogenin/Myf4 (Novocastra, Clone LO26) were newly performed. Immunostains for Ap2β (Santa Cruz Biotechnology, Rabbit Policlonal), and DAX-1 (mouse monoclonal 2F4) [12,13,19] were performed using an automated immunostainer. Ap2β and DAX-1 were considered positive when a strong nuclear staining was found in the majority of cells (S2 Table).

Rhabdomyosarcoma cell lines (HTB-82, RD, RH-30, RH-41 and RUCH-2) were cultured in DMEM (4.5 g/l glucose), supplemented with 10% FCS (Invitrogen) at 37°C in an atmosphere containing 5% CO_2_. Whole cell extracts were obtained by homogenizing the cells in hot Laemmli buffer and proteins were separated on SDS-PAGE and blotted on a polyvinyl difluoride membrane (GE Healthcare, Indianapolis, IN). Membranes were blocked and incubated with the primary antibodies, the anti-DAX-1 2F4 antibody [19] and the commercial anti β-tubulin antibody (Sigma-Aldrich) for antigen detection, overnight at 4°C. Membranes were incubated for 1 hour with horseradish peroxidase-conjugated antimouse or antirabbit antibody (Amersham Biosciences). Detection was performed using an enhanced chemiluminescence detection system from Roche on Kodak XK-1 films (Eastman Kodak Co., Rochester, NY) [20,21].

Chi-square test was performed in order to evaluate the correlation between DAX-1 pattern of expression and histology. Fisher’s exact test was performed in order to evaluate the association between the expression of DAX-1 and Ap2β.

Written consent for participation in the RMS 79 and RMS 96 studies had been requested and obtained at the moment of each patient’s registration in the protocol. The study was approved by the Ethics Committee of the University of Padua as part of the AIEOP (Italian Association of Pediatric Hematology-Oncology) clinical trials.

## Results

### Clinical features

Patients’ median age at diagnosis was 6.9 years (range 0–21). Tumor site was: limbs in 21, genito-urinary tract in 9, head-neck region in 12 (5 parameningeal, 7 non-parameningeal), and other sites in 26. Tumor size at radiological investigations varied from 1 to 20 cm (mean diameter 9.2). Stage of disease was available in 67 patients: 2 IRS I, 13 IRS II, 21 IRS III, and 31 IRS IV. Follow-up varied from 4 to 20 years (median 7.3). Twenty-nine patients died of disease.

### Pathological features

Seventy-one ARMS were re-classified as follow: 48 ARMS, 14 ERMS, 1 spindle cell/sclerosing RMS, 4 mixed RMS, 4 epithelioid RMS [22].

Among the 48 ARMS, 40 showed classic morphology with cellular monotony and only occasional rhabdomyoblastic differentiation. Four were solid and 4 showed histological features difficult to be classified; in fact one had a very tiny biopsy and cytologic detail was poorly preserved, another one displayed a solid pattern of growth of very primitive round cells without evidence of rhabdomyoblastic differentiation, and scattered enlarged, very atypical nuclei and in 2 cases cells were rather elongated. Forty-two tumors were translocation positive.

**Fig 1:**
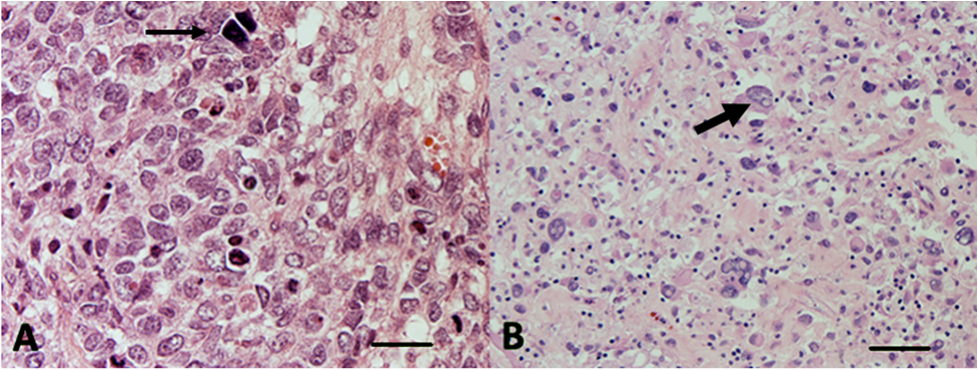
(A) Hematoxylin and Eosin stained section from a tumor with initial diagnosis of ARMS, based on the monotonous morphology with round polygonal cells with hypercromatic nuclei (thin arrow), embedded in a collagenized stroma (320x, scale bar 70 μm). (B) HE stained section from post-therapy specimen shows a reduction of cellularity with scattered cells displaying anaplastic features (thick arrow) with increased cell size (2-fold more than the adjacent cells) and nuclear hypercromasia. Atypical mitoses were seen in other areas (100x, scale bar 210 μm).

The group of tumors re-classified as ERMS included 5 cases of the so-called dense cellular type ERMS [11], characterized by a very dense cellularity and primitive morphology, and 9 anaplastic ERMS, characterized by an unusual cytology, with enlarged nuclei resembling the “nuclear unrest” of Wilms’ tumor and considered a precursor of anaplasia [4], together with evidence of scattered, frankly anaplastic cells (Fig 1).

The spindle cell/sclerosing RMS showed an abundant sclerohyaline stroma with evidence of nests and microalveoli mimicking classic alveoli of ARMS. There was no evidence of spindle cell areas.

In all ERMS and spindle cells/sclerosing RMS, molecular analysis did not show any specific translocation.

“Mixed” RMS were characterized by single or multiple nodules with features of classic ARMS embedded in an embryonal neoplasm: in one case the alveolar clusters were predominant, in the other, alveolar areas were mixed with embryonal, spindle cell, sclerosing or epithelioid areas.

Four epithelioid RMS, which are part of a recently published study by Zin *et al*., [22], were characterized by large epithelioid cells arranged in solid sheets, resembling a rhabdoid tumor.

All of the 23 “control” cases with the original diagnosis of ERMS showed the classic morphology.

### Immunohistochemistry

**Fig 2:**
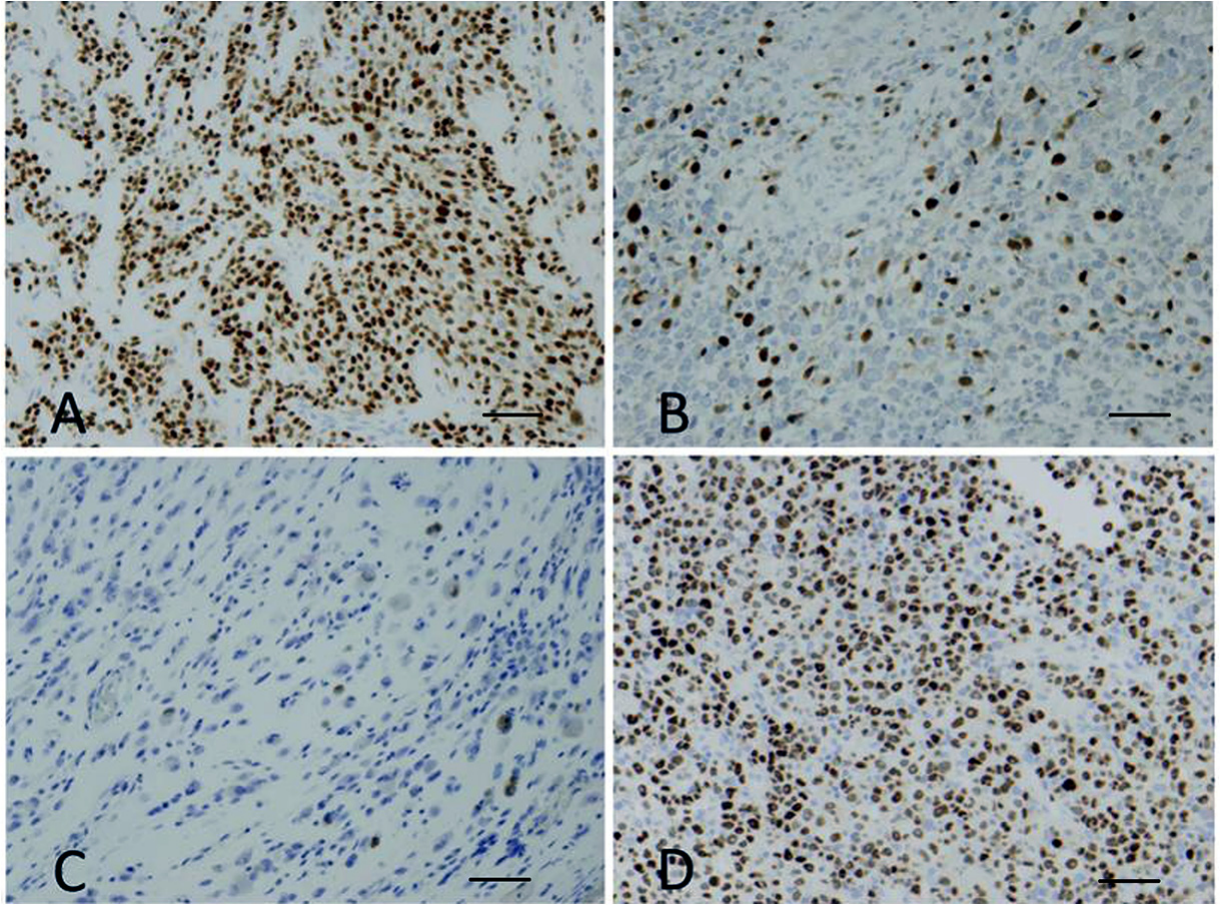
(A) Immunostaining for Myf4 in histological sections of a t +ARMS, showing a positive nuclear staining (brown cells) in more than 90% of cells. (B,C and D) Immunostaining for Myf4 in histological sections of a ERMS, immunostained for Myf4, varying from scattered cells (< 20%) to more than 40% and 70% of cells respectively (160x, scale bar 120 μm).

Results of immunostains are summarized in Table 1. All tumors showed a positive staining for desmin, ranging from strong and diffuse to focal. A strong positivity for Myf4 (Fig 2) in more than 50% of cells was detected in 84% of ARMS (37/43), 38.4% of tumors re-classified as ERMS (5/14), and 50% of epithelioid and mixed RMS. A weak expression of Myf4 was found in the single sclerosing RMS. Only 21% of classic ERMS (5/23) were diffusely positive for Myf4.

**Table 1:** Immunohistochemical pattern (according to histotype).

Reviewed Diagnosis (with translocation status)	Number of cases	#Myf4+/#cases (%)	#Ap2β+/#cases (%)	DAX-1+/#cases (%)
*ARMS (t+)*	42	32/37 (84)	26/32 (81)	29/33 (88)
*ARMS (t-)*	6	5/6 (83)	2/5 (40)	2/5 (40)
*ERMS (t-)*	14	5/14 (35)	0/14 (0)	3/14 (21)
*RMSm (t-)*	4	2/4 (50)	0/4 (0)	2/4 (75)
*RMSep (t-)*	4	2/4 (50)	0/4 (0)	1/2 (50)
*RMSscl (t-)*	1	0/1 (0)	0/1 (0)	1/1 (100)
*ERMS (controls*, *t-)*	*23 (100)*	*5/23 (21)*	*0/23 (0)*	*4/23 (17)*

ARMS: Alveolar Rhabdomyosarcoma; t+: translocation positive; t-: translocation negative; ERMS: Embryonal Rhabdomyosarcoma; RMSm: mixed Rhabdomyosarcoma; RMSep: epithelioid Rhabdomyosarcoma; RMSscl: sclerosing/spindle cell Rhabdomyosarcoma.

**Fig 3:**
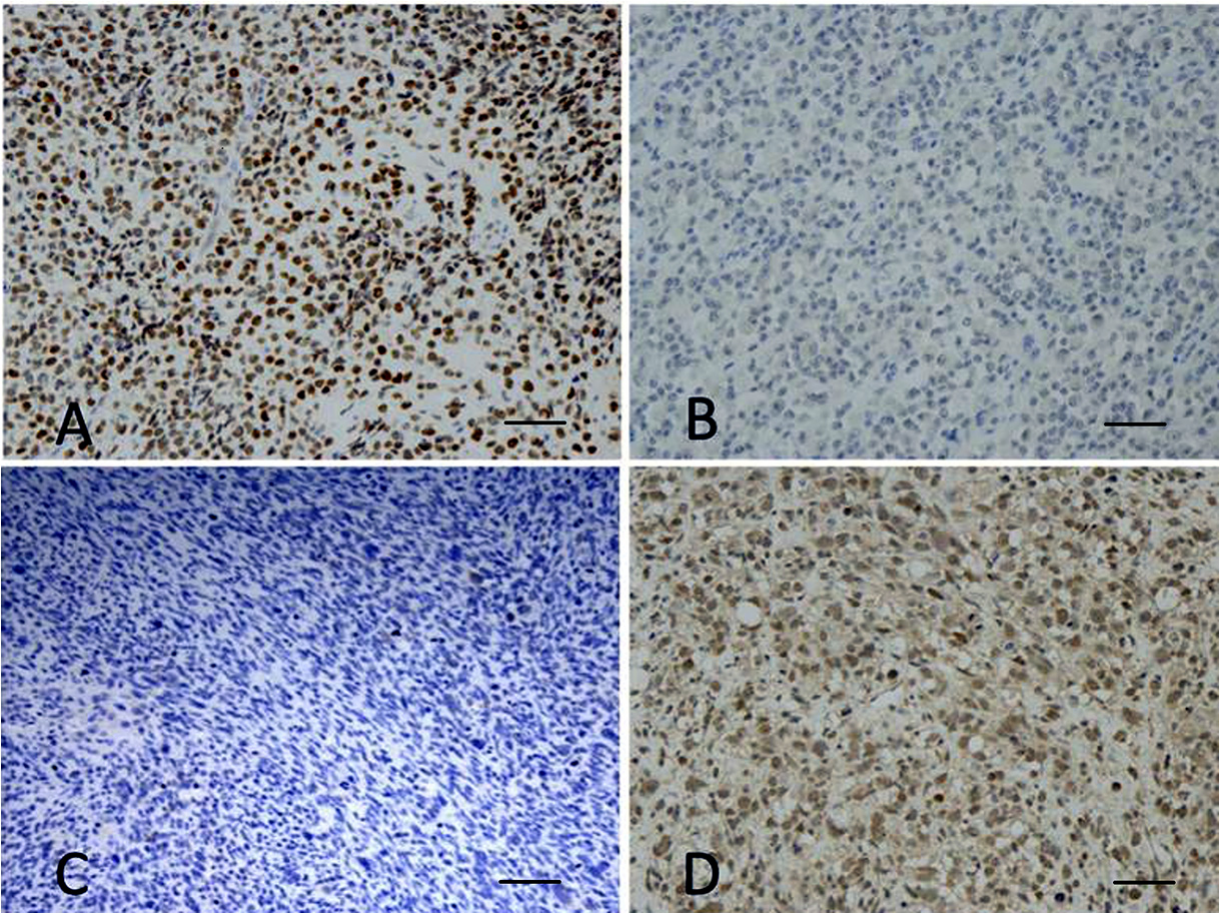
(A) Positive immunostaining for DAX-1 in histological section of a t+ ARMS. (B,C) Negative immunostaining for DAX-1 in histological sections of two different ERMS. (D) Immunostaining for DAX-1 in histological section of a ERMS with the typically weak staining found in non-ARMS (80x, scale bars 240 μm).

DAX-1 was expressed in 31/38 ARMS (81.5%; 29 t+ and 2 t-), in 3/14 cases re-classified as ERMS, in 50% of mixed RMS (2/4) and of epithelioid RMS (1/2), and in the sclerosing RMS. Four out of 23 (17%) classic ERMS were DAX-1 positive. Interestingly, only 4 out of the 8 RMS with non-classic morphology showed a strong and diffuse positive staining in more than 50% of cells, in contrast to the remaining tumors, which showed weak nuclear staining (Fig 3).

**Fig 4:**
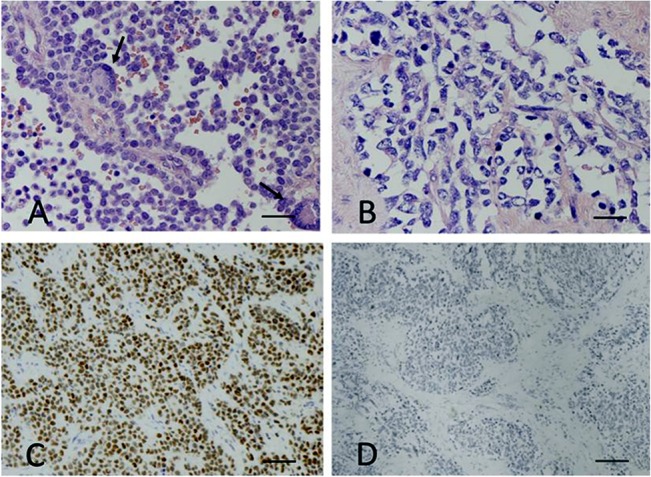
(A) Immunostaining for Ap2β in HE section of a t+ ARMS showing the typical alveolar pattern and wreath-like cells (thin arrow) (320x, scale bar 60 μm). (C) Immunostaining for Ap2β in HE section of a t+ ARMS, showing a diffuse nuclear staining in more than 90% of cells, in keeping with the presence of translocation (160x, scale bar 120 μm). (B) Mixed RMS; HE staining show an alveolar pattern, with polygonal cells lacking the monotony of an ARMS (320x scale bar 60 μm). (D) Negative Ap2β staining in HE section of the same mixed RMS confirming the lack of translocation (80x, scale bar 240 μm).

**Fig 5:**
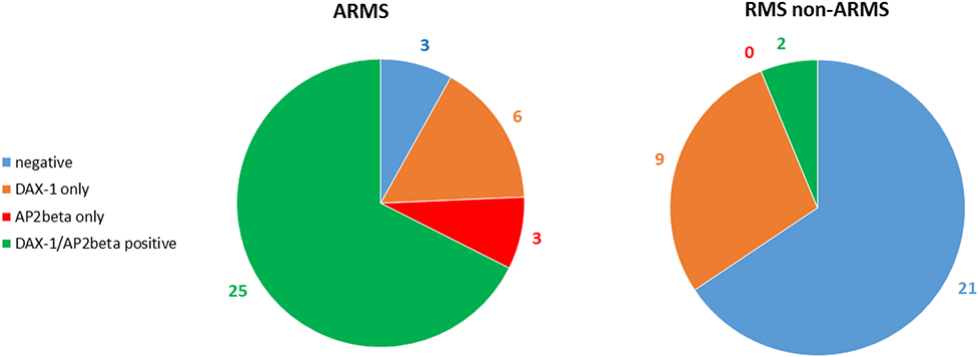
Pie charts showing the amounts of individual and co-expression of DAX-1 and Ap2β in ARMS t+ and non-ARMS. (The arabic numbers indicate the number of positive tumors for each marker).

All of the 14 new ERMS and 4 mixed RMS showed low or absent Ap2β expression. All 23 classic ERMS were negative for Ap2β, even in DAX-1-positive cases. Ap2β was expressed in 26/32 t+ ARMS and in 2/5 t- ARMS (Fig 4). The relationship between Ap2β and DAX-1 is summarized in Fig 5.

### DAX-1 expression in RMS cell lines

**Fig 6:**
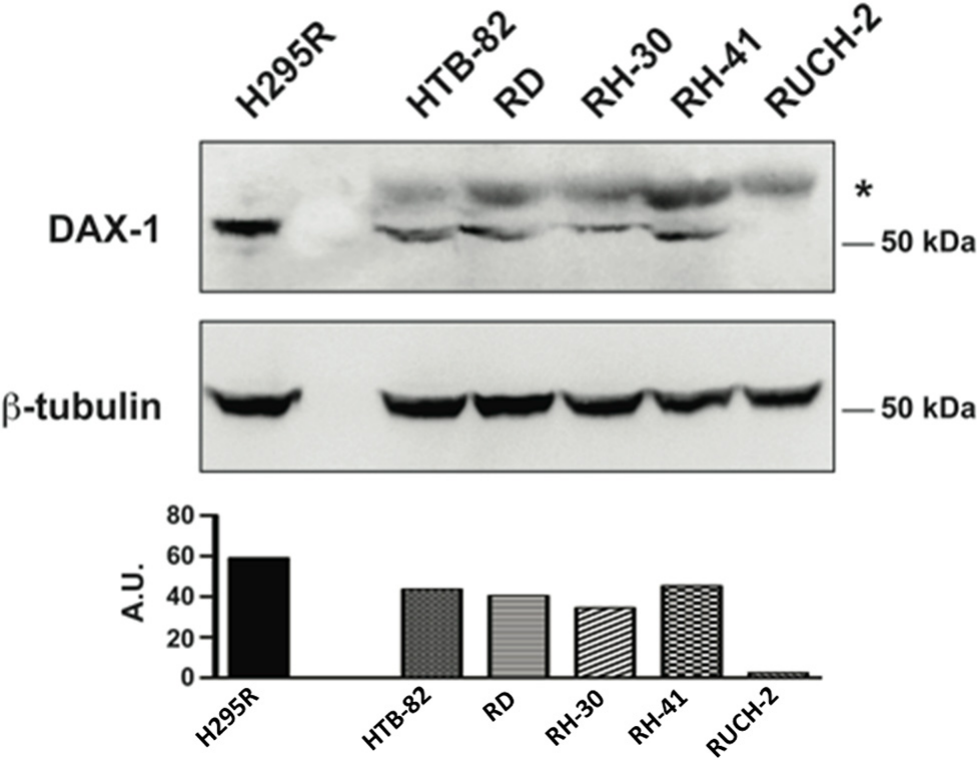
Western Blot showing DAX-1 expression in 4 out of five cell lines. The asterisk indicates a non-specific band present only in RMS cell lines. The histogram shows the relative amounts of DAX-1 expression in each cell line. The densitometry was calculated (ImageJ software) as the ratio between the intensities of the bands corresponding to DAX-1 for each cell line and the corresponding bands of β-tubulin multiplied by 100 and expressed in arbitrary units (AU).

DAX-1 expression was present in four out of five cell lines examined (Fig 6); in two ARMS cell lines (RH-30 and RH-41), in one ERMS cell line (HTB-82) and in one spindle cell/sclerosing RMS cell line (RD). Another ERMS cell line was negative (RUCH-2).

### Statistical analysis

DAX-1 immunohistochemical expression pattern in ARMS and case controls, constituted by classic ERMS, was significantly different (p < 0.003). Fisher’s exact test showed a significant association between the IHC expression of DAX-1 and Ap2β (p < 0.002).

## Discussion

The histological overlap between some ARMS and ERMS related to their morphologic heterogeneity [23,24,25,26,27,28,29,30] is one of the principal causes of the low diagnostic agreement (80.9% and 63.0% for ARMS and ERMS respectively) even among expert pathologists, as demonstrated by the IRSG-COG studies [17]. Recurrent translocations t(2;13)(q35,q13.1) and t(1,13)(p36;q13.1), detected in 50% and 25% of alveolar RMS, respectively, make the diagnosis of ARMS easier [16,31,32,33]. However it is not yet clear if rhabdomyosarcomas with alveolar morphology lacking the translocation belong to the group of ARMS and should be treated accordingly. Different reports on gene expression confirm the existence of a common molecular profile in t- ARMS and classic ERMS [34,35,36]. Moreover, a series of genes on the RNA level have been found to have a high specificity in the discrimination between these two subgroups, and might represent potential immunohistochemical markers. Myogenin is highly expressed in ARMS, but it is not a completely reliable marker in the differential diagnosis between ARMS and ERMS, since its expression is highly variable in ERMS; conversely AP2β (*TFAP2*) has been found to be selectively expressed in t+ ARMS and might represent a helpful tool in the morphologic diagnosis. [10,11,37].

The present series well underlines the difficulty in the morphologic diagnosis of ARMS and ERMS if we consider that only 48 out of 71 tumors initially diagnosed as ARMS were confirmed. The major diagnostic difficulties were encountered in ERMS with dense cellularity and primitive cytology, as already demonstrated [11], and in those RMS with a peculiar morphology not fitting in the classic categories, such as epithelioid RMS and RMS with “nuclear unrest”, i.e. with enlarged nuclei but not sufficient to define them as anaplastic. Sclerosing RMS is frequently characterized by microalveolar areas that can be misinterpreted as classic alveoli of ARMS. As reported in the recent WHO classification, mixed RMS, considered in the past as ARMS, are probably ERMS, as confirmed by the absence of translocation in our cases [2].

Ap2β (*TFAP2*) immunostains are sensitive (81%) and highly specific (96%) in the detection of t+ ARMS. The low sensitivity, as already reported by Wachtel *et al*., [10], may be related to the inadequate antigenic preservation due, in some cases, to a prolonged or inadequate fixation. Interestingly, 23 cases with an initial diagnosis of ARMS, and re-classified as ERMS, spindle cell/sclerosing RMS and mixed RMS, were Ap2β negative. Among these cases myogenin immunostains were strongly and diffusely positive in 9.

DAX-1 (*NR0B1*) is an orphan nuclear receptor playing a key role in the development of adrenal gland [38,39]. Its expression in mouse embryonic stem cells is controlled by the transcription factor Nanog [40]. The immunohistochemical expression of DAX-1 has been reported to correlate with the presence of mRNA, evaluated with quantitative RT-PCR, confirming IHC as a valid evaluation tool [41]. Recent studies demonstrated that DAX-1 is abundantly expressed by embryonic stem cells in an undifferentiated status and its level decreases with differentiation. Knock-down of DAX-1 expression results in differentiation of embryonic stem cells, suggesting a role of DAX-1 for the maintenance of undifferentiated state [42,43]. These features support a potential role of DAX-1 in tumorigenesis: in fact, it has been found to be expressed in several adult malignancies, including endometrial carcinoma, ovarian carcinoma, prostatic carcinoma and lung adenocarcinoma. Moreover, recent reports demonstrated that DAX-1 can be regulated by EWS-FLI1 and is required for the transformed phenotype of Ewing sarcoma cells [13,15,41], promoting cell proliferation. However, the role of DAX-1 in deregulation of developmental pathways in pediatric cancer has not been further investigated. DAX-1 expression in ARMS has been evaluated only in one study, which showed positivity in 2 cases of translocation-positive ARMS, but not in two cases of ERMS [13].

In the present series 29/33 t+ ARMS showed a strong and diffuse positive immunostaining for DAX-1. DAX-1 immunostaining was more sensitive (88%), but less specific (41%) compared to Ap2β. About 60% of RMS initially diagnosed as ARMS and reclassified as other subtypes of RMS, except ERMS, showed a diffuse positivity, although only rarely comparable to that of t+ ARMS; while, in control ERMS and in ERMS formerly diagnosed as ARMS, DAX-1 was present in 17% and 0% respectively. These data are confirmed by Western Blot analysis that showed the presence of DAX-1 in 4/5 RMS cell lines (2/2 alveolar, 1/2 embryonal and 1/1 spindle cell/sclerosing). Moreover they also suggest that DAX-1 and alveolar morphology are significantly associated and that this marker may be helpful in the distinction of classic ARMS and ERMS. However, in the group of RMS with mixed features or in epithelioid and sclerosing RMS, DAX-1 may be frequently expressed and should be studied in combination with Ap2β and Myogenin. In fact, their combined use concurred to identify most of t+ ARMS (strong positivity of all three markers in 22, two in 3 and only one in 4 cases), while 9/17 RMS re-classified as non-ARMS were negative for all markers. Remarkably, in Ewing sarcoma cells, the fusion transcript EWS-FLI1 promotes directly DAX-1 expression, which regulates pathways involved in cell growth and proliferation [14]: high DAX-1 expression in t+ ARMS may similarly correlate with genomic regulation mechanism triggered by fusion transcripts. However further studies are needed to investigate the relationship between DAX-1 and stem cell markers, in particular Nanog 1, which is involved in the regulation of DAX-1 for the control of the stem cell status.

In conclusion, as clearly stated in recent studies, the diagnosis of ARMS requires a molecular confirmation, and Ap2β is a reliable immunohistochemical marker of t+ ARMS. Our study demonstrates that DAX-1 is significantly associated with t+ ARMS and may be a useful adjunct to Ap2β although less specific than Ap2β. As reported in other studies, the morphologic spectrum of translocation negative tumors, previously called ARMS on the basis of morphology only, is wide and perhaps includes different subtypes characterized also by a higher expression of DAX-1 compared to classic ERMS. However, the expression of DAX-1 in a subset of RMS discloses new fascinating perspectives concerning its role in preventing cell differentiation and promoting replication in analogy to the pathogenic mechanisms demonstrated in Ewing sarcoma. New insights in the role of DAX-1 rhabdomyosarcoma tumorigenesis may lead to a future development of novel targeted therapies [12].

## Supporting Information

S1 Table RT-PCR: primer and probe. FAM-TAMRA pairs were used for Taqman probes.
(DOC)

S2 Table Immunohistochemistry: antibodies and concentrations. (DOC)

S1 Fig Western Blot showing DAX-1 expression in 4 out of five cell lines (original unadjusted and uncropped blot). (TIF)

S2 Fig Western blot for β-tubulin as control (original unadjusted and uncropped blot). (TIF)
