# Supplementary material for: DAX-1 Expression in Pediatric Rhabdomyosarcomas: Another Immunohistochemical Marker Useful in the Diagnosis of Translocation Positive Alveolar Rhabdomyosarcoma
Source: PLoS One. 2015 Jul 13;10(7):e0133019. doi: 10.1371/journal.pone.0133019 (PMC4500404; doi:10.1371/journal.pone.0133019)
Supplement: S1 Table — FAM-TAMRA pairs were used for Taqman probes. (DOC) [file pone.0133019.s001.doc]

**S1 Table. RT-PCR: primer and probe.**

| ***Transcribed*** |  | ***Sequence*** | ***Amplicon***  ***(bp)*** |
| --- | --- | --- | --- |
| ABL | **Forward**  **Reverse**  **Probe** | 5’-CAACACTGCTTCTGATGGCAA-3’  5’-CGGCCACCGTTGAATGAT-3’  5’-CAACACCCTGGCCGAGTTGGTTCAT–3’ | 92 |
| PAX3-FKHR | **Forward**  **Reverse**  **Probe** | 5’-TGAACCCCACCATTGGCAAT-3’  5’-CTGTGTAGGGACAGATTATGACGAA-3’  5’-TGGCCTCTCACCTCAGAATTCAATTCGT–3’ | 67 |
| PAX7-FKHR | **Forward**  **Reverse**  **Probe** | 5’-GGTCAGCAACGGCCTGTCT-3’  5’-CATTCTGCACACGAATGAACTTG-3’  5’-CTCAGGAATTCAATTCGTCATAATCTGTCCCTACA–3’ | 80 |

FAM-TAMRA pairs were used for Taqman probes.
