# Supplementary material for: DAX-1 Expression in Pediatric Rhabdomyosarcomas: Another Immunohistochemical Marker Useful in the Diagnosis of Translocation Positive Alveolar Rhabdomyosarcoma
Source: PLoS One. 2015 Jul 13;10(7):e0133019. doi: 10.1371/journal.pone.0133019 (PMC4500404; doi:10.1371/journal.pone.0133019)
Supplement: S2 Table — (DOC) [file pone.0133019.s002.doc]

**S2 Table. Immunohistochemistry: antibodies and techniques**

| **Antibody** | **Manufacturer** | **Clone** | **Dilution*** | **Retrieval** | **Positive controls** |
| --- | --- | --- | --- | --- | --- |
| Myf-4 | Novocastra | Clone LO26 | 1:20 | Sodium citrate (30’, 100°C) | RMS |
| Desmin | Dako Cytomation | Clone D33 | 1:100 | None | RMS |
| Ap2β | Santa Cruz Biotechnology | Rabbit polyclonal | 1:70 | EDTA  (30’, 100°C) | ARMS t+ |
| DAX-1 | E. Lalli | Mouse monoclonal 2F4 | 1:300 | Sodium citrate (30’, 100°C) | ARMS |

***** Bond Primary Antibody Diluent, Leica
