# Supplementary figures and images for: DAX-1 Expression in Pediatric Rhabdomyosarcomas: Another Immunohistochemical Marker Useful in the Diagnosis of Translocation Positive Alveolar Rhabdomyosarcoma
Source: PLoS One. 2015 Jul 13;10(7):e0133019. doi: 10.1371/journal.pone.0133019 (PMC4500404; doi:10.1371/journal.pone.0133019)

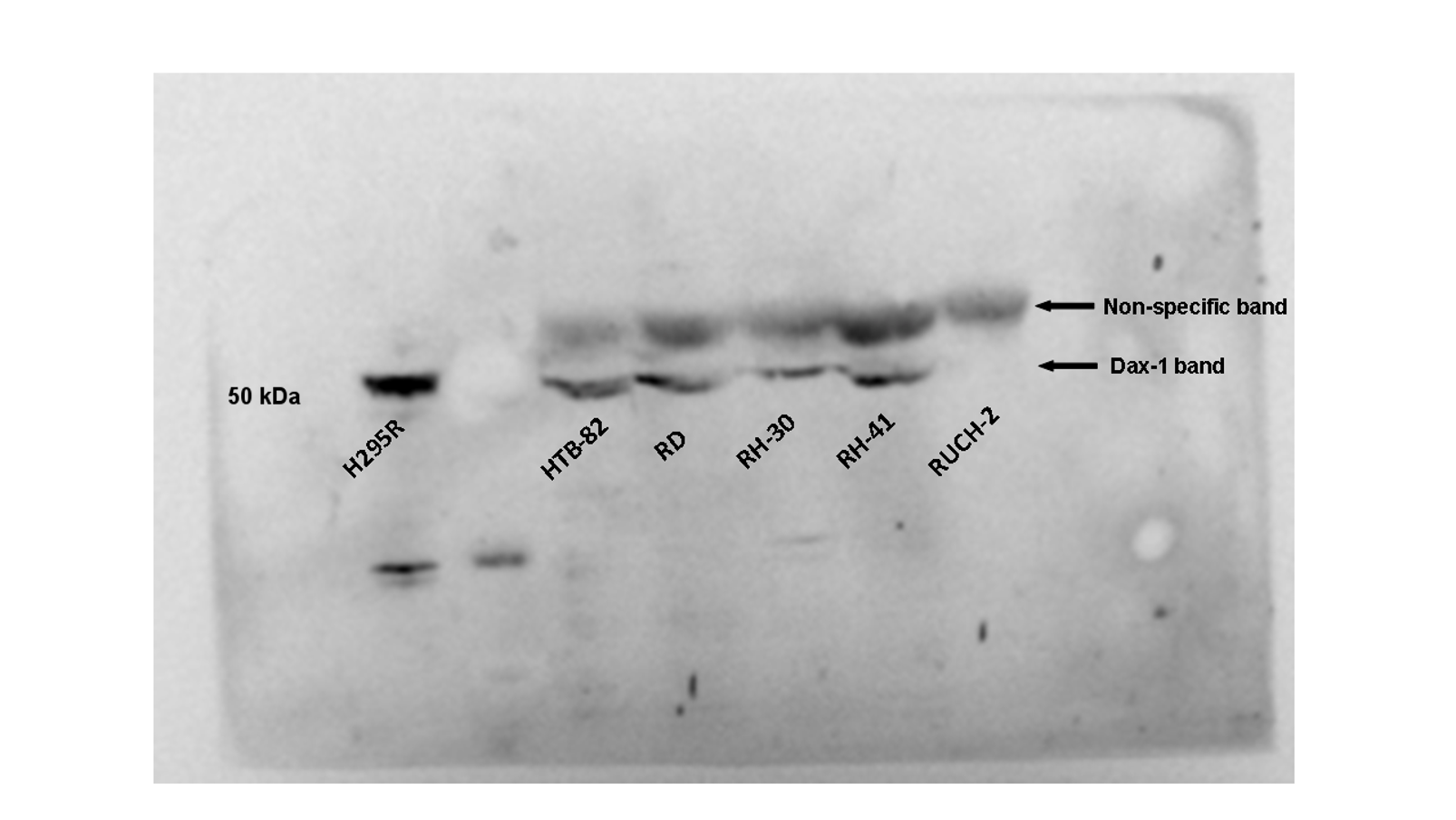

Supplement: S1 Fig — (TIF) [file pone.0133019.s003.tif]

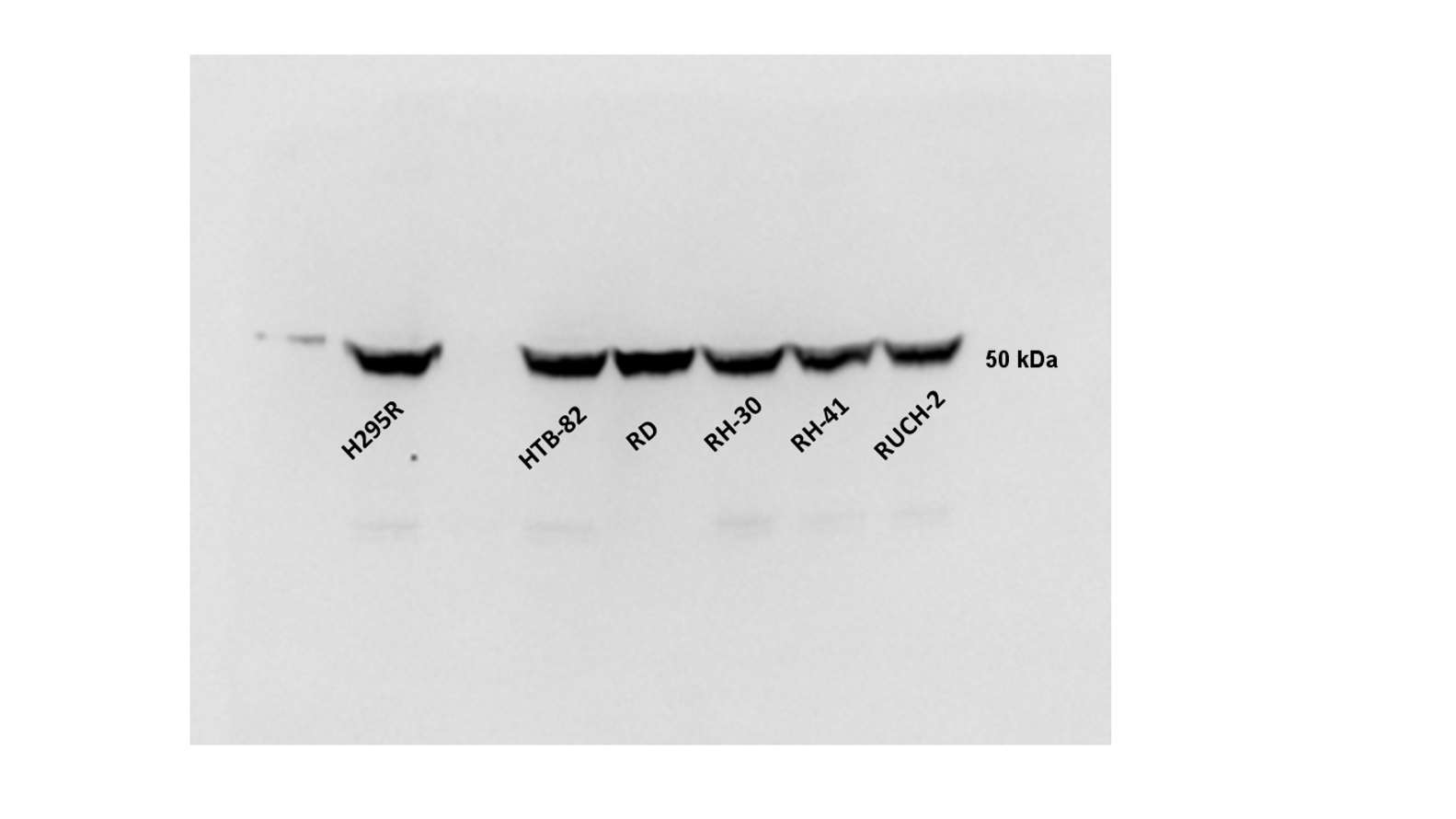

Supplement: S2 Fig — (TIF) [file pone.0133019.s004.tif]
